# Abdominal aortic calcification in patients with CKD

**DOI:** 10.1007/s40620-015-0260-7

**Published:** 2016-03-22

**Authors:** Mieke J. Peeters, Jan AJG van den Brand, Arjan D. van Zuilen, Yelka Koster, Michiel L. Bots, Marc G. Vervloet, Peter J. Blankestijn, Jack FM Wetzels, For the MASTERPLAN Study Group

**Affiliations:** 1 464 Department of Nephrology, Radboud University Medical Center, PO box 9101, 6500 HB Nijmegen, The Netherlands; 2 Department of Nephrology, University Medical Center Utrecht, Utrecht, The Netherlands; 3 Julius Center for Health Sciences and Primary Care, University Medical Center Utrecht, Utrecht, The Netherlands; 4 Department of Nephrology, VU University Medical Center, Amsterdam, The Netherlands

**Keywords:** Abdominal aortic calcification, Cardiovascular risk, Chronic kidney disease, Prognosis

## Abstract

**Background:**

Abdominal aortic calcification (AAC) is independently associated with cardiovascular events in dialysis patients and in the general population. However, data in non-dialysis chronic kidney disease (CKD) patients are limited. We analyzed determinants and prognostic value of AAC in non-dialysis CKD patients.

**Methods:**

We included patients with CKD not receiving renal replacement therapy from the MASTERPLAN study, a randomized controlled trial that started in 2004. In the period 2008–2009, an X-ray to evaluate AAC was performed in a subgroup of patients. We studied AAC using a semi-quantitative scoring system by lateral lumbar X-ray. We used baseline and 2-year data to find determinants of AAC. We used a composite cardiovascular endpoint and propensity score matching to evaluate the prognostic value of AAC.

**Results:**

In 280 patients an X-ray was performed. In 79 patients (28 %) the X-ray showed no calcification, in 62 patients (22 %) calcification was minor (<4), while 139 patients (50 %) had moderate or heavy calcification (≥4). Older age, prior cardiovascular disease, higher triglyceride levels, and higher phosphate levels were independent determinants of a calcification score ≥4. AAC score ≥4 was independently associated with cardiovascular events, with a hazard ratio of 5.5 (95 % confidence interval 1.2–24.8).

**Conclusions:**

Assessment of AAC can identify CKD patients at higher cardiovascular risk, and may provide important information for personalized treatment. Whether this approach will ultimately translate into better outcomes remains to be answered.

**Electronic supplementary material:**

The online version of this article (doi:10.1007/s40620-015-0260-7) contains supplementary material, which is available to authorized users.

## Introduction

Patients with chronic kidney disease (CKD) are at increased risk of cardiovascular events [1]. Disturbances in bone and mineral metabolism play an important role. For example, hyperparathyroidism, hypercalcemia, hyperphosphatemia, and elevated fibroblast growth factor 23 (FGF23) are associated with cardiovascular morbidity and mortality [2, 3]. It has been suggested that disturbances in bone and mineral metabolism cause vascular calcification [4, 5]. Vascular calcification, either in the coronary arteries or in the aorta, is related to cardiovascular events in dialysis patients and in the general population [6–10]. The 2009 Kidney Disease: Improving Global Outcomes (KDIGO) clinical practice guideline on CKD mineral and bone disorder (CKD-MBD) suggests using plain radiographs to evaluate abdominal aortic calcification (AAC) in selected patients in order to assist in personalized treatment advice [11]. However, data on the prognostic value of AAC in non-dialysis CKD patients are limited [12, 13]. Therefore, we studied the severity, determinants, and prognostic value of AAC in non-dialysis CKD patients.

## Methods

### Design and patient selection

The MASTERPLAN (Multifactorial Approach and Superior Treatment Efficacy in Renal Patients with the Aid of Nurse practitioners) study was a randomized controlled trial that evaluated the added value of nurse practitioner care in reducing cardiovascular events and attenuating kidney function decline in patients with prevalent CKD [ISRCTN registry number 73187232]. Its rationale, design and outcomes have been published elsewhere [14–16]. Ethics committee approval was obtained for the study as well as written informed consent from all participants. Patients were included in the study between April 2004 and December 2005, and followed thereafter. Although specific treatment goals were defined, routine patient care was left to the discretion of the treating practitioner. In the period 2008–2009 nephrologists considered the role of evaluating AAC in selected patients, based on the data and discussions that resulted in the recommendation in the 2009 KDIGO CKD-MBD guideline [11]. The MASTERPLAN steering committee at that time decided that performing a lateral lumbar X-ray was not part of the study protocol, but the decision whether to use it in patient care was left to the treating nephrologist.

We evaluated the use of the lateral lumbar X-ray in CKD patients that participated in the MASTERPLAN study. We included non-transplanted patients with a lateral lumbar X-ray in 2008–2009 who did not develop end-stage renal disease (ESRD) before the X-ray was taken. For comparison, we also selected all non-transplanted patients without an X-ray in 2008–2009 who did not develop ESRD before 2008.

### Assessment of AAC

We reviewed all lateral lumbar X-rays. For evaluation of AAC, we used a semi-quantitative scoring system, as described by Kauppila et al. [17]. Briefly, the abdominal aorta adjacent to the first four lumbar vertebrae was divided into four segments using the midpoint of each intervertebral space as a boundary. Anterior and posterior aortic wall segments were evaluated separately. Calcific deposits were graded on a scale of 0–3 at each segment, as follows: 0 = no calcific deposits, 1 = small scattered calcific deposits filling less than one-third of the aortic wall, 2 = one-third to two-thirds of the aortic wall calcified, 3 = at least two-thirds of the aortic wall calcified. The grades of the eight aortic segments were summed in the Kauppila calcification score (the antero-posterior severity score), ranging from 0 to 24 points. Two independent observers (MP and YK) scored all lateral lumbar X-rays. Both observers were blinded to the clinical and laboratory patient data.

### Cardiovascular outcome

As described before, follow-up in the MASTERPLAN study was extended for the analysis of renal endpoints [16]. We retrieved information on mortality and renal outcome parameters from the participating centers. At the same time, we collected additional data on cardiovascular events. In this study we used a composite cardiovascular outcome of myocardial infarction, coronary artery bypass grafting, percutaneous coronary intervention (PCI), stroke, percutaneous treatment of peripheral arterial disease (PTA), bypass of peripheral arteries, amputation, treatment of aortic aneurysm, treatment of renal artery stenosis, and cardiovascular mortality.

### Statistical analyses

We compared baseline characteristics of patients with an X-ray vs. patients without an X-ray using independent-samples T test, Mann–Whitney U test, and Chi square test where appropriate. We calculated the linearly weighted Kappa to evaluate inter-rater agreement [18]. We used the mean scores attributed by the two observers in the subsequent analyses.

For continuous variables, we used mean values of MASTERPLAN baseline characteristics and those at 2 years to represent the period before the lateral lumbar X-ray. We considered a categorical variable present when it was present either at baseline or at 2 years. We imputed missing data by multiple imputation before mean values were computed [19]. Fifty imputed datasets were created. At baseline, data for nine variables were missing with missing percentages of 0.4–10.4 % per variable. At 2-year follow-up, almost all variables had missing data with missing percentages of 1.8–18.2 % per variable.

We tabulated patient characteristics, expressed as a percentage for categorical, and mean ± standard deviation (SD), or median and interquartile range (IQR) for continuous variables, by low (<4, no or minor calcification) vs. high calcification score (≥4, moderate or heavy calcification). Differences between the groups were studied by logistic regression. On the basis of our previous analyses, we expected a low cardiovascular event rate [15]. Since the study population for the current analyses was quite small, we chose to primarily dichotomize AAC into groups of equal size. This decision also seemed reasonable, given that other investigators have also divided their population on the basis of median AAC [20, 21].

Next, we used multivariate logistic regression to identify independent determinants of AAC.

We performed Cox regression to analyze univariate relationships between patient characteristics and cardiovascular events. Because of the low incidence of cardiovascular events, and therefore the limited number of predictors that could be included in multivariate Cox regression analysis, we used propensity score matching to determine whether AAC may add prognostic value beyond known predictive factors for cardiovascular events [22, 23].

Using a multivariate logistic regression model including known predictors of cardiovascular events, we estimated the probability of a high calcification score (≥4). This is the propensity score. Among patients with a similar propensity score, some in actual fact have a high and others a low calcification score. Matching two patients with similar propensity scores (one with a high and one with a low calcification score) yields pairs of patients who are comparable in terms of cardiovascular risk factors, except for the calcification score. If a difference in cardiovascular outcome is subsequently observed, it indicates that AAC has prognostic value over and above the traditional risk factors.

We included the following clinical risk factors (mainly on the basis of the Framingham Risk Score [24]) in the multivariate logistic regression model: age, gender, history of diabetes mellitus, prior cardiovascular disease, systolic blood pressure, low-density lipoprotein (LDL) cholesterol, high-density lipoprotein (HDL) cholesterol, smoking status, antihypertensive drug use, triglyceride and phosphate levels. Propensity scores were estimated for all imputed datasets, the average for every patient was used for propensity score matching [25]. A caliper distance of 0.01 was used. In this matched sample, we performed Kaplan–Meier analysis and Cox regression stratified on the matched pairs.

We performed two sensitivity analyses. First, we evaluated absence versus presence of calcification in addition to the analyses comparing patients with low and high calcification score. Second, to evaluate the influence of AAC on therapeutic decision making, we compared mean clinical parameters and medication use of the two visits after the X-ray to characteristics in the period before the X-ray was taken.

All p-values were two-sided, and a p < 0.05 was considered to indicate statistical significance. Analyses were performed using IBM SPSS Statistics Version 20 (IBM Corporation, Armonk, NY, USA). Cox regression stratified on matched pairs was performed using Stata 11.2 (StataCorp LP, College Station, TX, USA).

## Results

We evaluated lateral lumbar X-rays taken in the period 2008–2009. In 7 out of 9 centers participating in MASTERPLAN, X-rays were performed. The percentage of patients with an X-ray ranged from 26 to 65 % per center. In total, we included in this study 280 patients with available lateral lumbar X-rays. For comparison purposes, we used data of patients without an X-ray who were followed in the 7 centers. The patients with an X-ray were randomized to the intervention group, adhered to the physical activity guideline, and used aspirin more often than patients without an X-ray. Furthermore, patients with an X-ray had a higher ankle brachial index, lower protein creatinine ratio, higher HDL cholesterol, lower phosphate, and lower FGF23 levels (Supplementary Table 1). The lateral lumbar X-rays were taken within a median of 3.7 years [IQR 3.1–4.0] of baseline.

### Assessment and severity of AAC

**Fig. 1:**
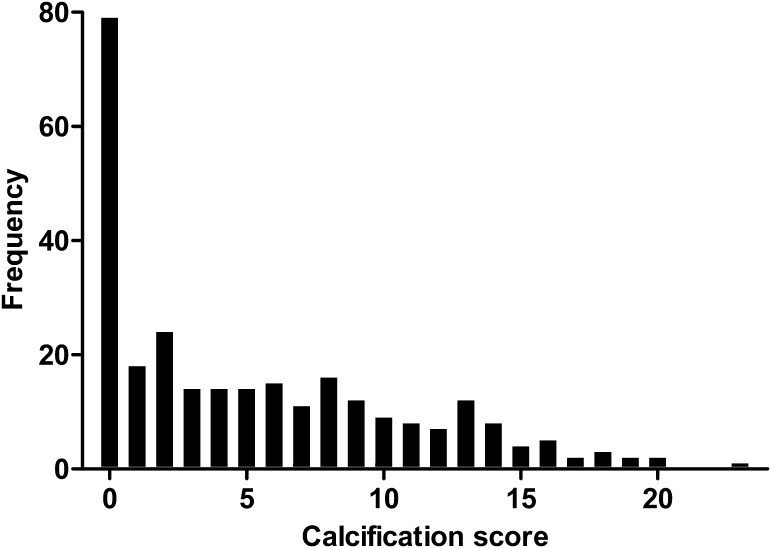
Severity of abdominal aortic calcification (X-rays, n = 280). The median calcification score was 3.5, interquartile range 0–8.9

Inter-rater agreement was very good with a linearly weighted Kappa of 0.87 (Supplementary Data 1) [18]. Supplementary Fig. 1 shows the frequencies of calcification scores attributed by the two observers. The frequency distribution, using the mean scores of the two observers, is illustrated in Fig. 1. The median calcification score was 3.5 [IQR 0–8.9]. In 79 patients (28 %) the X-ray showed no AAC. Calcification was more prominent in the lower segments of the aorta. Furthermore, calcification scores were higher in the posterior wall compared to the anterior wall (Supplementary Fig. 2).

### Determinants of AAC

Table 1 shows characteristics of patients with a low calcification score (<4) compared to patients with a high calcification score (≥4). The patients who had a higher calcification score were older, more often had a history of diabetes mellitus (and higher HbA1c levels) and cardiovascular disease. Furthermore, a higher calcification score was associated with higher systolic blood pressure, triglyceride and phosphate levels, and lower ankle brachial index. Patients with a high calcification score used a statin and antihypertensive drugs more often than patients with a low calcification score. Estimated glomerular filtration rate (eGFR) did not differ between the two groups. In multivariate analysis, older age, prior cardiovascular disease, higher triglyceride levels, and higher phosphate levels were independent determinants of a high calcification score (Table 2).

**Table 1:** Characteristics of patients by calcification score

Characteristic	Calcification score		p
	<4 (n = 141)	≥4 (n = 139)	
Randomized to intervention group	61 %	56 %	0.41
Age (years)	55.2 (12.9)	65.9 (8.3)	<0.001
Male gender	67 %	70 %	0.66
Caucasian race	89 %	90 %	0.88
Renovascular cause of kidney disease	28 %	35 %	0.22
History of diabetes mellitus^a^	20 %	33 %	0.01
Prior cardiovascular disease^b^	18 %	42 %	<0.001
eGFR^c^ (ml/min/1.73 m^2^)	36.6 (12.0)	36.3 (13.3)	0.81
Systolic blood pressure (mmHg)	132 (15)	138 (18)	0.004
Diastolic blood pressure (mmHg)	80 (10)	78 (10)	0.12
Ankle brachial index^d^	1.12 (0.17)	1.07 (0.20)	0.01
Protein creatinine ratio (mg/10 mmol)	123 [27–472]	121 [18–646]	0.17
Total cholesterol (mmol/l)	4.64 (0.85)	4.63 (0.82)	0.92
LDL cholesterol (mmol/l)	2.61 (0.81)	2.56 (0.70)	0.56
HDL cholesterol (mmol/l)	1.39 (0.42)	1.31 (0.39)	0.10
Triglycerides (mmol/l)	1.56 (0.83)	1.81 (0.98)	0.03
Calcium (mmol/l)	2.36 (0.11)	2.36 (0.12)	0.74
Phosphate (mmol/l)	1.07 (0.19)	1.14 (0.20)	0.003
PTH (pmol/l)	7.5 [5.6–11.8]	8.3 [5.5–12.0]	0.66
FGF23 (RU/ml)	99 [63–161]	134 [70–191]	0.14
Hemoglobin (mmol/l)	8.4 (0.9)	8.3 (0.8)	0.55
Serum albumin (g/l)	40.5 (3.4)	39.7 (3.1)	0.04
HbA1c (%)	5.9 (0.8)	6.2 (0.7)	<0.001
BMI (kg/m^2^)	26.2 (4.8)	27.1 (3.8)	0.09
Smoking	22 %	21 %	0.85
Urinary sodium creatinine ratio (mmol/mmol)	13.9 (4.6)	14.1 (4.8)	0.72
Physical activity guideline adherence	79 %	78 %	0.82
Aspirin use	57 %	64 %	0.22
Oral anticoagulant drug use	11 %	15 %	0.33
Statin use	87 %	95 %	0.02
Vitamin D use	41 %	42 %	0.87
Antihypertensive drug use	93 %	99 %	0.04
Calcium containing phosphate binder use	12 %	10 %	0.50
Sevelamer use	6 %	6 %	0.95
Erythropoiesis stimulating agent use	16 %	15 %	0.90

Studied by logistic regression. Mean values of baseline and data at 2 years were used

Data are given as percentage, mean (SD), or median [interquartile range]

*eGFR* estimated glomerular filtration rate, *LDL* low density lipoprotein, *HDL* high density lipoprotein, *PTH* parathyroid hormone, *FGF23* fibroblast growth factor 23, *BMI* body mass index

^a^Diabetes mellitus is defined as using blood glucose lowering medication or fasting glucose >7.0 mmol/l

^b^Cardiovascular disease is defined as myocardial infarction, stroke, or vascular intervention

^c^Using the MDRD equation re-expressed for standardized serum creatinine

^d^Measurement from the leg with the lower ankle brachial index was used

**Table 2:** Multivariate analysis, independent determinants of a high calcification score

Characteristic	OR	95 % CI	p
Age/10 (years)	2.53	1.88–3.41	<0.001
Prior cardiovascular disease^a^	2.42	1.30–4.50	0.01
Triglycerides/0.1 (mmol/l)	1.04	1.01–1.08	0.01
Phosphate/0.1 (mmol/l)	1.29	1.10–1.50	0.001

Studied by multivariate logistic regression

Nagelkerke R^2^ = 0.35–0.37

*OR* odds ratio, *CI* confidence interval

^a^Cardiovascular disease is defined as myocardial infarction, stroke, or vascular intervention

### Cardiovascular outcome

Median follow-up duration after the lateral lumbar X-ray was 2.4 years. A cardiovascular event occurred in 6 out of 141 patients with a low calcification score and in 20 out of 139 patients with a high calcification score (≥4). The 26 cardiovascular events included: myocardial infarction (7 patients), PCI (4 patients), stroke (3 patients), PTA (5 patients), bypass of peripheral arteries (1 patient), aortic aneurysm (2 patients), and cardiovascular mortality (4 patients).

Older age, renovascular cause of kidney disease, lower eGFR, higher systolic blood pressure, higher protein creatinine ratio, lower calcium, higher FGF23, lower hemoglobin, lower serum albumin levels, and oral anticoagulant drug use were associated with the composite cardiovascular outcome at univariate Cox regression analysis. The use of aspirin was related to a lower cardiovascular event rate. Moreover, a calcification score ≥4 was associated with cardiovascular events (Table 3).

**Table 3:** Associations with cardiovascular outcome in univariate Cox regression

Characteristic	HR	95 % CI	p
Calcification score ≥4	3.86	1.55–9.62	0.004
Randomized to intervention group	0.53	0.24–1.14	0.11
Age/10 (years)	2.11	1.36–3.28	0.001
Male gender	1.05	0.46–2.41	0.92
Caucasian race	1.38	0.33–5.84	0.66
Renovascular cause of kidney disease	2.53	1.17–5.47	0.02
History of diabetes mellitus^a^	1.04	0.44–2.48	0.93
Prior cardiovascular disease^b^	1.50	0.68–3.32	0.31
eGFR^c^/5 (ml/min/1.73 m^2^)	0.83	0.69–0.99	0.04
Systolic blood pressure/10 (mmHg)	1.53	1.22–1.91	<0.001
Diastolic blood pressure/10 (mmHg)	1.04	0.70–1.55	0.83
Ankle brachial index^d^/0.1	0.89	0.74–1.06	0.19
Ln protein creatinine ratio (Ln of mg/10 mmol)	1.21	1.00–1.46	0.049
Total cholesterol/0.1 (mmol/l)	1.02	0.98–1.07	0.38
LDL cholesterol/0.1 (mmol/l)	1.02	0.97–1.07	0.46
HDL cholesterol/0.1 (mmol/l)	0.96	0.86–1.07	0.42
Triglycerides/0.1 (mmol/l)	1.03	1.00–1.07	0.07
Calcium/0.1 (mmol/l)	0.60	0.41–0.88	0.01
Phosphate/0.1 (mmol/l)	1.06	0.88–1.27	0.56
Ln PTH (Ln of pmol/l)	1.45	0.74–2.87	0.28
Ln FGF23 (Ln of RU/ml)	1.89	1.26–2.82	0.002
Hemoglobin (mmol/l)	0.56	0.34–0.93	0.02
Serum albumin (g/l)	0.87	0.78–0.97	0.01
HbA1c (%)	1.30	0.83–2.04	0.25
BMI (kg/m^2^)	1.00	0.91–1.09	0.94
Smoking	1.09	0.44–2.74	0.85
Urinary sodium creatinine ratio/0.1 (mmol/mmol)	1.00	0.99–1.01	0.52
Physical activity guideline adherence	1.19	0.43–3.32	0.74
Aspirin use	0.40	0.18–0.88	0.02
Oral anticoagulant drug use	2.53	1.06–6.06	0.04
Statin use	0.82	0.25–2.75	0.75
Vitamin D use	1.46	0.68–3.15	0.34
Antihypertensive drug use	0.47	0.11–1.98	0.30
Calcium containing phosphate binder use	0.32	0.04–2.38	0.27
Sevelamer use^e^	–	–	–
Erythropoiesis stimulating agent use	1.03	0.35–3.00	0.96

*HR* hazard ratio, *CI* confidence interval, *eGFR* estimated glomerular filtration rate, *LDL* low density lipoprotein, *HDL* high density lipoprotein, *PTH* parathyroid hormone, *FGF23* fibroblast growth factor 23, *BMI* body mass index

^a^Diabetes mellitus is defined as using blood glucose lowering medication or fasting glucose >7.0 mmol/l

^b^Cardiovascular disease is defined as myocardial infarction, stroke, or vascular intervention

^c^Using the MDRD equation re-expressed for standardized serum creatinine

^d^Measurement from the leg with the lower ankle brachial index was used

^e^Adequate Cox regression was not possible, since there were no cardiovascular events in the patients who used sevelamer

**Fig. 2:**
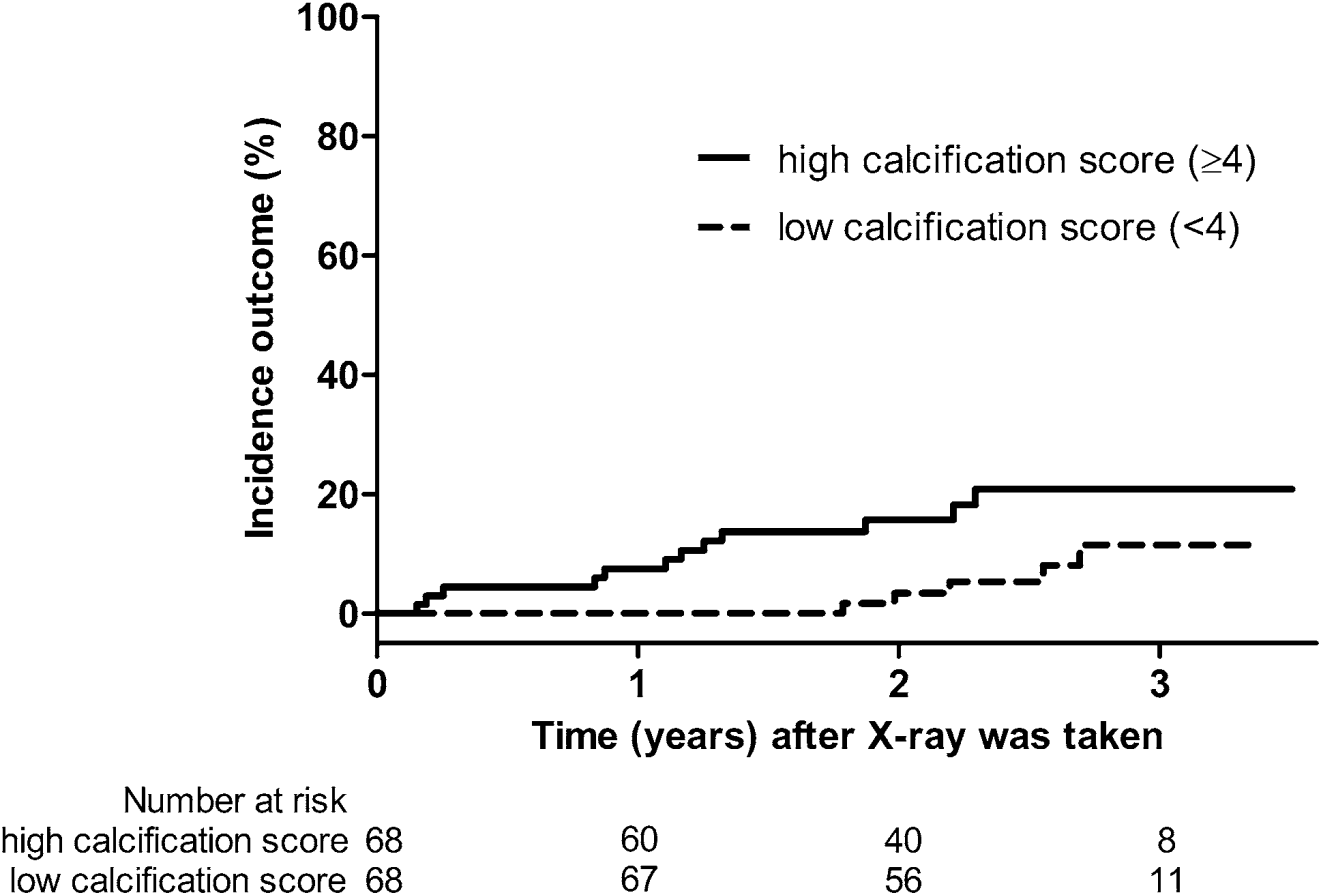
Incidence of the composite cardiovascular outcome in propensity score matched patients. Stratified on matched pairs, p = 0.03

Multivariate Cox regression could not be performed reliably, because of the low incidence of cardiovascular events. Therefore, a propensity score matched sample was used to evaluate whether a high calcification score added prognostic value to known cardiovascular risk factors. Sixty-eight matched pairs of patients with low and high calcification score were included. Cardiovascular risk factors were balanced between the two groups (Supplementary Table 2). In the 68 patients with a low calcification score, 5 had a cardiovascular event. In 12 out of 68 patients with a high calcification score a cardiovascular event occurred. The hazard ratio (HR) for cardiovascular events in the high calcification score group was 5.5 (95 % confidence interval 1.2–24.8), p = 0.03. The Kaplan–Meier curves are shown in Fig. 2.

### Sensitivity analyses

The analyses evaluating absence versus presence of calcification (calcification score 0 vs. >0) yielded similar results to the analyses comparing patients with low and high calcification score (Supplementary Tables 3 and 4, and Supplementary Data 2). We did not observe major differences in treatment changes after the X-ray between patients with low vs. high calcification scores. The only differences we observed were a slight increase in phosphate binder use in patients with a high calcification score, and a small decrease in beta blocker use in patients with a low calcification score (data not shown).

## Discussion

In this study, we showed that: (1) AAC was a common occurrence in our population of non-dialysis CKD patients; (2) older age, prior cardiovascular disease, higher triglyceride levels, and higher phosphate levels were independent determinants of a high calcification score; and, most importantly, (3) AAC had prognostic value for cardiovascular events in non-dialysis CKD patients.

### Prevalence of AAC

AAC was common in our population of non-dialysis CKD patients. Presence of AAC was found in 72 % of patients. This is similar to results in other CKD populations [26, 27]. Decades ago, in fact, it was demonstrated that vascular calcification is more extended and more severe in patients with CKD than in age-matched healthy individuals [28]. Also, vascular calcification increases gradually with progressing CKD [29]. Using lateral lumbar X-rays, in dialysis patients an AAC prevalence of up to 94 % has been described, with duration of dialysis being independently associated with severity of AAC [30]. For comparison, in the Framingham Heart Study, a general population cohort, 67 % of 1030 men (mean age 60.4 years), and 58 % of 1437 women (mean age 60.8 years) showed AAC on their X-ray [31]. Also in a randomly selected sample of men from the region of Lyon, France (STRAMBO cohort), in 780 men aged ≥60 years (mean age 72 years) median Kauppila calcification score was 1 [IQR 0–4], and 41 % showed no aortic calcification [21]. In a study of healthy living kidney donors, AAC was detected by computed tomography (CT) in only 31 % [32].

### Determinants of AAC

In our population, older age, prior cardiovascular disease, higher triglyceride levels, and higher phosphate levels were independent determinants of a high calcification score.

Age was the most important determinant of vascular calcification. In CKD patients, dialysis patients, as well as in the general population, a direct relationship between age and AAC has been consistently observed [21, 26, 27, 30, 33, 34]. In addition, it has frequently been observed that patients with (severe) AAC more often have a cardiovascular disease history [26, 30, 33, 34]. Evidence on the role of disturbances in bone and mineral metabolism in the development of AAC is not consistent. It is well known that vascular calcification is not just a passive process of calcium and phosphate deposition due to serum supersaturation. It is an active, complex, and dynamically regulated process, resulting in phenotypical transformation of vascular smooth muscle cells into osteoblast-like cells [35]. Although it seems obvious that, for instance, plasma phosphate level plays an important role in this process [5, 36], it has not always been identified as a risk factor for AAC in clinical studies [27, 30, 37]. Nevertheless, it is likely that parameters of CKD-MBD interact at the patient level to promote vascular calcification [11]. Recent data point to concerns about excessive calcium intake, including the use of calcium containing phosphate binders, with regard to progression of cardiovascular calcification [38]. This will be one of the issues reconsidered in the KDIGO CKD-MBD guideline update [39]. Furthermore, on the role of triglyceride (or other lipid) levels, results are contradictory [34, 37, 40]. It is well known that LDL cholesterol plays a critical role in atherosclerosis. Besides, it is recognized that triglycerides, or rather the lipoproteins that they are associated with, promote atherogenesis independently of LDL cholesterol [41]. The majority of patients in our study used a statin. Although statins have a triglyceride lowering effect, their effect on LDL cholesterol is larger [42]. Therefore, triglycerides may better reflect the lipid profile prior to statin use that contributed to the development of AAC. In addition, hypertriglyceridemia is in general the most common dyslipidemia in CKD [43] and might therefore be more strongly associated with AAC.

In studies using CT, eGFR proved an independent determinant of AAC in non-dialysis CKD patients [12, 44]. Studies using plain X-rays have not always confirmed this association [37, 40]. Also in our analyses, eGFR was not associated with AAC. A factor that probably contributed to this finding is that there was a difference in eGFR between patients with and without a cardiovascular disease history: eGFR was 39.4 and 35.1 ml/min/1.73 m^2^ in patients with and without prior cardiovascular disease respectively (p = 0.01). Since cardiovascular disease history was an important determinant of AAC, it possibly masked the effect of eGFR. We did not observe a relationship between eGFR and other determinants of calcification.

### Cardiovascular outcome

To the best of our knowledge, we are the first to show that the Kauppila calcification score is associated with cardiovascular events in non-dialysis CKD patients. This indicates that a lateral lumbar X-ray may provide information that can aid in clinical decision making.

Imaging markers like AAC are often better outcome predictors than serum markers, because they carry different prognostic information. Cardiovascular calcification represents the cumulative result of prolonged exposure to multiple risk factors, whereas serum markers only reflect the risk at the time of measurement [45].

It was already known that the Kauppila calcification score is independently related to cardiovascular morbidity and mortality in the general population and in dialysis patients [9, 10]. However, the association between vascular calcification and cardiovascular events in non-dialysis CKD patients has been scantly addressed. In non-dialysis CKD patients it was known that both coronary artery calcification [46] and AAC assessed by CT [12] are independently related to cardiovascular events, that presence of polyvascular calcification is associated with cardiovascular mortality [47], and that AAC quantified by the Kauppila calcification score correlates with coronary artery calcification [48].

### Strengths and limitations of our study

A lateral lumbar X-ray to assess severity of AAC is not as sensitive as other modalities such as CT [45]. Therefore we may have underestimated the severity of AAC in our population. However, lateral lumbar X-rays also have important advantages over CT: they are relatively inexpensive, involve low exposure to radiation, and are widely available and easy to use in daily clinical practice.

Other investigators have shown good to excellent inter-rater agreement on the Kauppila calcification score [17, 21, 26, 30, 34]. In these studies summary scores were used to test inter-rater agreement. We used individual segment scores, which are more accurate than summary scores. In the other studies, the X-rays were often scored by experienced radiologists. Although the two observers in our study (MP and YK) were not radiologists, we also established a very good inter-rater agreement. This is an important finding, since it implies that the semi-quantitative scoring system, described by Kauppila et al. [17], is indeed a simple imaging technique that can be readily used by clinicians after minimal training.

Several laboratory parameters that are important in the context of vascular calcification were not available, such as serum vitamin D and fetuin-A levels. Moreover, AAC could be the result of prolonged low-grade inflammation [49]. In this context highly sensitive C-reactive protein (hsCRP) is important. In MASTERPLAN, hsCRP values were only available at baseline, and therefore we did not include this parameter in our analysis. However, we did study hsCRP at baseline. Median levels were 1.62 mg/l [IQR 0.61–4.15] and 1.90 mg/l [IQR 0.93–5.13] in the low and high calcification group, respectively (p = 0.17).

Unfortunately, data on dosage and duration of medication use were not available.

Another limitation of our study is that the patients enrolled were participating in a clinical trial, and X-rays were performed in a subgroup. In patients who participated in the MASTERPLAN study, risk factor levels were already quite well controlled at baseline [16]. Therefore, the studied patients may not be representative of the CKD population in general, compromising the study’s external validity.

When compared to various other CKD cohorts, the mortality rate in MASTERPLAN is among the lowest in the world [50]. In this study, the cardiovascular event rate was low during a limited follow-up. We used propensity score matching instead of the traditional regression model to circumvent problems of overfitting, and were able to demonstrate the prognostic value of AAC by lateral lumbar X-ray in non-dialysis CKD patients.

## Conclusion

Our study supports the recommendation in the KDIGO guideline that assessment of AAC in CKD patients can identify patients at higher cardiovascular risk and may provide important information for personalized treatment. Whether this approach will ultimately translate into better outcomes, however, remains to be answered.

## Electronic supplementary material

Below is the link to the electronic supplementary material.

Supplementary material 1 (DOC 183 kb)
