# Supplementary material for: Abdominal aortic calcification in patients with CKD
Source: J Nephrol. 2016 Mar 22;30(1):109–18. doi: 10.1007/s40620-015-0260-7 (PMC5316387; doi:10.1007/s40620-015-0260-7)
Supplement: Supplementary file 1 — Supplementary material 1 (DOC 183 kb) [file 40620_2015_260_MOESM1_ESM.doc]

**Supplementary material:**

*Page 2*

**Supplementary Table 1** Baseline characteristics of patients with and without a lateral lumbar X-ray in 2008-2009

*Page 4*

**Supplementary Data 1** Calculation of weighted Kappa (κ_w_)

*Page 5*

**Supplementary Fig. 1** Frequencies of calcification scores by the two observers (280 X-rays)

*Page 6*

**Supplementary Fig. 2** Mean calcification score per aortic segment (280 X-rays)

*Page 7*

**Supplementary Table 2** Characteristics of the propensity score matched patients

*Page 8*

**Supplementary Table 3** Characteristics of patients by absence versus presence of calcification

*Page 9*

**Supplementary Table 4** Multivariate analysis, independent determinants of presence of calcification (calcification score >0)

*Page 10*

**Supplementary Data 2** Cardiovascular events in patients with or without calcification

*Page 11*

**Reference**

**Supplementary Table 1** Baseline characteristics of patients with and without a lateral lumbar X-ray in 2008-2009

| **Characteristic** | **Patients with lateral**  **lumbar X-ray (n=280)** | **Patients without lateral**  **lumbar X-ray (n=175)^a^** | **P- value** |
| --- | --- | --- | --- |
| Randomized to intervention group | 59% | 39% | <0.001 |
| Age (years) | 59.5 (12.1) | 60.6 (14.2) | 0.39 |
| Male gender | 69% | 65% | 0.38 |
| Caucasian race | 90% | 93% | 0.29 |
| Nephrological diagnosis |  |  | 0.62 |
| Diabetic nephropathy | 11% | 10% |  |
| Renovascular | 31% | 31% |  |
| Glomerulonephritis | 19% | 17% |  |
| Interstitial nephritis | 11% | 14% |  |
| Congenital | 9% | 13% |  |
| Unknown | 20% | 15% |  |
| History of diabetes mellitus^b^ | 23% | 25% | 0.68 |
| Prior cardiovascular disease^c^ | 29% | 29% | 0.96 |
| eGFR^d^ (ml/min/1.73m^2^) | 37.1 (12.7) | 35.5 (14.1) | 0.19 |
| Serum creatinine (µmol/l) | 170 (58) | 181 (72) | 0.09 |
| Systolic blood pressure (mmHg) | 136 (20) | 138 (21) | 0.26 |
| Diastolic blood pressure (mmHg) | 79 (12) | 80 (11) | 0.66 |
| Ankle brachial index^e^ | 1.10 (0.19) | 1.02 (0.20) | <0.001 |
| Protein creatinine ratio (mg/10 mmol) | 150 [41-668] | 304 [55-977] | 0.01 |
| Total cholesterol (mmol/l) | 4.88 (1.07) | 4.85 (1.09) | 0.74 |
| LDL cholesterol (mmol/l) | 2.83 (0.98) | 2.84 (0.91) | 0.88 |
| HDL cholesterol (mmol/l) | 1.35 (0.42) | 1.21 (0.37) | <0.001 |
| Triglycerides (mmol/l) | 1.78 (1.11) | 1.99 (1.39) | 0.08 |
| Calcium (mmol/l) | 2.38 (0.14) | 2.35 (0.14) | 0.10 |
| Phosphate (mmol/l) | 1.10 (0.24) | 1.15 (0.28) | 0.03 |
| PTH (pmol/l) | 8.1 [5.2-13.0] | 8.3 [4.8-13.1] | 0.84 |
| FGF23 (RU/ml) | 97 [54-162] | 116 [67-182] | 0.046 |
| Hemoglobin (mmol/l) | 8.3 (0.9) | 8.2 (1.0) | 0.17 |
| Serum albumin (g/l) | 40.5 (3.7) | 39.8 (4.0) | 0.05 |
| HbA1c (%) | 6.1 (0.8) | 6.3 (1.0) | 0.05 |
| BMI (kg/m^2^) | 26.6 (4.5) | 26.9 (4.5) | 0.43 |
| Current smoking | 19% | 24% | 0.21 |
| Urinary sodium creatinine ratio (mmol/mmol) | 13.7 (5.0) | 14.3 (5.8) | 0.29 |
| Physical activity guideline adherence | 64% | 54% | 0.04 |
| Aspirin use | 42% | 27% | 0.001 |
| Oral anticoagulant drug use | 11% | 14% | 0.47 |
| Statin use | 71% | 65% | 0.13 |
| Vitamin D use | 18% | 23% | 0.16 |
| Antihypertensive drug use | 94% | 94% | 0.93 |
| Calcium containing phosphate binder use | 6% | 9% | 0.28 |
| Sevelamer use | 3% | 4% | 0.66 |
| Erythropoiesis stimulating agent use | 8% | 9% | 0.68 |

Studied by independent-samples T test, Mann-Whitney U test, or chi-square test where appropriate.

Abbreviations: eGFR, estimated glomerular filtration rate; LDL, low density lipoprotein; HDL, high density lipoprotein; PTH, parathyroid hormone; FGF23, fibroblast growth factor 23; BMI, body mass index.

Data are given as percentage, mean (SD), or median [interquartile range].

^a^Only patients from the seven centers where lateral lumbar X-rays were taken were included.

^b^Diabetes mellitus is defined as using blood glucose lowering medication or fasting glucose > 7.0 mmol/l.

^c^Cardiovascular disease is defined as myocardial infarction, stroke, or vascular intervention.

^d^Using the MDRD equation reexpressed for standardized serum creatinine.

^e^Measurement from the leg with the lower ankle brachial index was used.

**Supplementary Data 1** Calculation of weighted Kappa (κ_w_)

280 lateral lumbar X-rays * 8 segments = 2240 segments

|  |  | Observer 1 | | | |  |
| --- | --- | --- | --- | --- | --- | --- |
|  | Calcification score  per segment | 0 | 1 | 2 | 3 | Total observer 2 |
| Observer 2 | 0 | 1360  (0.6071) | 45  (0.0201) | 3  (0.0013) | 0 | 1408  (0.6286) |
|  | 1 | 51  (0.0228) | 304  (0.1357) | 28  (0.0125) | 1  (0.0004) | 384  (0.1714) |
|  | 2 | 4  (0.0018) | 56  (0.025) | 167  (0.0746) | 14  (0.0063) | 241  (0.1076) |
|  | 3 | 0 | 0 | 53  (0.0237) | 154  (0.0688) | 207  (0.0924) |
|  | Total observer 1 | 1415  (0.6317) | 405  (0.1808) | 251  (0.1121) | 169  (0.0754) | 2240  (1) |

Weight matrix

|  |  | Observer 1 | | | |  |
| --- | --- | --- | --- | --- | --- | --- |
|  | Calcification score  per segment | 0 | 1 | 2 | 3 |  |
| Observer 2 | 0 | 1 | 0.6667 | 0.3333 | 0 |  |
|  | 1 | 0.6667 | 1 | 0.6667 | 0.3333 |  |
|  | 2 | 0.3333 | 0.6667 | 1 | 0.6667 |  |
|  | 3 | 0 | 0.3333 | 0.6667 | 1 |  |
|  |  |  |  |  |  |  |

P_0_ = 0.6071*1 + 0.0201*0.6667 + 0.0013*0.3333 + 0 +

0.0228*0.6667 + 0.1357*1 + 0.0125*0.6667 + 0.0004*0.3333 +

0.0018*0.3333 + 0.025*0.6667 + 0.0746*1 + 0.0063*0.6667 +

0 + 0 + 0.0237*0.6667 + 0.0688*1 = 0.961

P_e_ = 1*(0.6286*0.6317) + 0.6667*(0.6286*0.1808) + 0.3333*(0.6286*0.1121) + 0 +

0.6667*(0.1714*0.6317) + 1*(0.1714*0.1808) + 0.6667*(0.1714*0.1121) + 0.3333*(0.1714*0.0754) +

0.3333*(0.1076*0.6317) + 0.6667*(0.1076*0.1808) + 1*(0.1076*0.1121) + 0.6667*(0.1076*0.0754) +

0 + 0.3333*(0.0924*0.1808) + 0.6667*(0.0924*0.1121) + 1*(0.0924*0.0754) = 0.689

κ_w_ = (P_0_ - P_e_) / (1 - P_e_) = (0.961 - 0.689) / (1 - 0.689) = 0.272 / 0.311 = 0.87

**Supplementary Fig. 1** Frequencies of calcification scores by the two observers (280 X-rays)

**
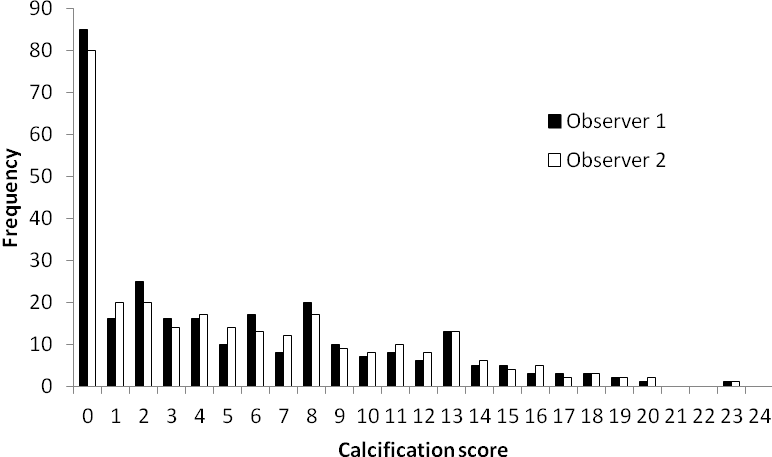
**

**Supplementary Fig. 2** Mean calcification score per aortic segment (280 X-rays)


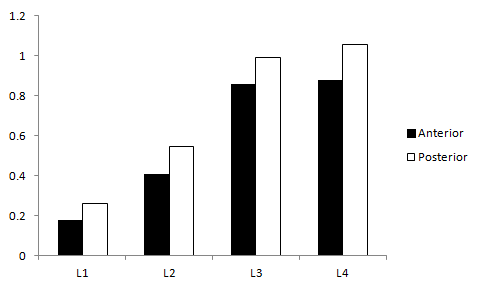


**Supplementary Table 2** Characteristics of the propensity score matched patients

|  | **Calcification score** | |  |
| --- | --- | --- | --- |
| **Characteristic** | **<4**  **(n=68)** | **≥4**  **(n=68)** | **P-value** |
| Randomized to intervention group | 65% | 53% | 0.17 |
| Age (years) | 62.5 (9.1) | 62.8 (8.2) | 0.85 |
| Male gender | 69% | 78% | 0.25 |
| Caucasian race | 90% | 90% | 1.00 |
| Renovascular cause of kidney disease | 31% | 34% | 0.71 |
| History of diabetes mellitus^a^ | 28% | 26% | 0.77 |
| Prior cardiovascular disease^b^ | 26% | 29% | 0.70 |
| eGFR^c^ (ml/min/1.73m^2^) | 35.5 (12.0) | 35.5 (13.0) | 0.99 |
| Systolic blood pressure (mmHg) | 137 (15) | 136 (19) | 0.81 |
| Diastolic blood pressure (mmHg) | 80 (11) | 79 (10) | 0.44 |
| Ankle brachial index^d^ | 1.12 (0.16) | 1.09 (0.20) | 0.26 |
| Protein creatinine ratio (mg/10 mmol) | 128 [20-582] | 131 [21-854] | 0.85 |
| Total cholesterol (mmol/l) | 4.70 (0.79) | 4.58 (0.83) | 0.38 |
| LDL cholesterol (mmol/l) | 2.58 (0.79) | 2.53 (0.69) | 0.70 |
| HDL cholesterol (mmol/l) | 1.39 (0.42) | 1.32 (0.40) | 0.37 |
| Triglycerides (mmol/l) | 1.74 (0.94) | 1.65 (0.91) | 0.57 |
| Calcium (mmol/l) | 2.35 (0.11) | 2.36 (0.10) | 0.48 |
| Phosphate (mmol/l) | 1.11 (0.18) | 1.08 (0.16) | 0.26 |
| PTH (pmol/l) | 7.7 [5.4-12.5] | 9.5 [6.3-12.2] | 0.33 |
| FGF23 (RU/ml) | 112 [65-174] | 135 [69-174] | 0.65 |
| Hemoglobin (mmol/l) | 8.3 (0.9) | 8.3 (0.8) | 0.81 |
| Serum albumin (g/l) | 40.3 (3.3) | 39.9 (3.4) | 0.48 |
| HbA1c (%) | 6.0 (0.8) | 6.1 (0.7) | 0.45 |
| BMI (kg/m^2^) | 26.0 (4.4) | 26.1 (3.3) | 0.89 |
| Current smoking | 19% | 23% | 0.65 |
| Urinary sodium creatinine ratio (mmol/mmol) | 14.0 (5.2) | 13.7 (4.0) | 0.69 |
| Physical activity guideline adherence | 83% | 77% | 0.41 |
| Aspirin use | 55% | 60% | 0.61 |
| Oral anticoagulant drug use | 12% | 14% | 0.64 |
| Statin use | 87% | 95% | 0.13 |
| Vitamin D use | 41% | 38% | 0.75 |
| Antihypertensive drug use | 97% | 100% | 1.00 |
| Calcium containing phosphate binder use | 10% | 6% | 0.39 |
| Sevelamer use | 6% | 4% | 0.66 |
| Erythropoiesis stimulating agent use | 12% | 17% | 0.37 |

Studied by logistic regression.

Data are given as percentage, mean (SD), or median [interquartile range].

Abbreviations: eGFR, estimated glomerular filtration rate; LDL, low density lipoprotein; HDL, high density lipoprotein; PTH, parathyroid hormone; FGF23, fibroblast growth factor 23; BMI, body mass index.

^a^Diabetes mellitus is defined as using blood glucose lowering medication or fasting glucose > 7.0 mmol/l.

^b^Cardiovascular disease is defined as myocardial infarction, stroke, or vascular intervention.

^c^Using the MDRD equation reexpressed for standardized serum creatinine.

^d^Measurement from the leg with the lower ankle brachial index was used.

**Supplementary Table 3** Characteristics of patients by absence versus presence of calcification

|  | **Calcification score** | |  |
| --- | --- | --- | --- |
| **Characteristic** | **0**  **(n=79)** | **>0**  **(n=201)** | **P-value** |
| Randomized to intervention group | 58% | 59% | 0.94 |
| Age (years) | 51.5 (14.0) | 64.1 (9.1) | <0.001 |
| Male gender | 65% | 70% | 0.37 |
| Caucasian race | 86% | 91% | 0.22 |
| Renovascular cause of kidney disease | 22% | 35% | 0.03 |
| History of diabetes mellitus^a^ | 13% | 32% | 0.001 |
| Prior cardiovascular disease^b^ | 14% | 37% | <0.001 |
| eGFR^c^ (ml/min/1.73m^2^) | 36.2 (11.5) | 36.5 (13.1) | 0.86 |
| Systolic blood pressure (mmHg) | 129 (15) | 137 (17) | 0.001 |
| Diastolic blood pressure (mmHg) | 79 (9) | 79 (10) | 0.50 |
| Ankle brachial index^d^ | 1.13 (0.17) | 1.08 (0.19) | 0.07 |
| Protein creatinine ratio (mg/10 mmol) | 162 [41-607] | 105 [15-547] | 0.04 |
| Total cholesterol (mmol/l) | 4.71 (0.94) | 4.60 (0.79) | 0.32 |
| LDL cholesterol (mmol/l) | 2.65 (0.90) | 2.56 (0.69) | 0.40 |
| HDL cholesterol (mmol/l) | 1.39 (0.42) | 1.34 (0.40) | 0.36 |
| Triglycerides (mmol/l) | 1.66 (0.94) | 1.69 (0.91) | 0.85 |
| Calcium (mmol/l) | 2.37 (0.12) | 2.36 (0.11) | 0.31 |
| Phosphate (mmol/l) | 1.07 (0.19) | 1.12 (0.20) | 0.045 |
| PTH (pmol/l) | 8.0 [5.7-12.4] | 7.9 [5.3-11.8] | 0.52 |
| FGF23 (RU/ml) | 101 [63-180] | 119 [68-175] | 0.92 |
| Hemoglobin (mmol/l) | 8.2 (0.9) | 8.4 (0.8) | 0.17 |
| Serum albumin (g/l) | 40.7 (3.6) | 39.9 (3.2) | 0.07 |
| HbA1c (%) | 5.8 (0.8) | 6.2 (0.7) | <0.001 |
| BMI (kg/m^2^) | 25.6 (4.6) | 27.0 (4.2) | 0.02 |
| Current smoking | 19% | 22% | 0.57 |
| Urinary sodium creatinine ratio (mmol/mmol) | 13.9 (5.1) | 14.0 (4.6) | 0.91 |
| Physical activity guideline adherence | 82% | 77% | 0.43 |
| Aspirin use | 52% | 64% | 0.08 |
| Oral anticoagulant drug use | 9% | 15% | 0.18 |
| Statin use | 82% | 94% | 0.004 |
| Vitamin D use | 39% | 42% | 0.66 |
| Antihypertensive drug use | 90% | 98% | 0.01 |
| Calcium containing phosphate binder use | 10% | 11% | 0.79 |
| Sevelamer use | 6% | 6% | 0.88 |
| Erythropoiesis stimulating agent use | 15% | 16% | 0.89 |

Studied by logistic regression.

Data are given as percentage, mean (SD), or median [interquartile range].

Abbreviations: eGFR, estimated glomerular filtration rate; LDL, low density lipoprotein; HDL, high density lipoprotein; PTH, parathyroid hormone; FGF23, fibroblast growth factor 23; BMI, body mass index.

^a^Diabetes mellitus is defined as using blood glucose lowering medication or fasting glucose > 7.0 mmol/l.

^b^Cardiovascular disease is defined as myocardial infarction, stroke, or vascular intervention.

^c^Using the MDRD equation reexpressed for standardized serum creatinine.

^d^Measurement from the leg with the lower ankle brachial index was used.

**Supplementary Table 4** Multivariate analysis, independent determinants of presence of calcification (calcification score >0)

| **Characteristic** | **OR** | **95% CI** | **P-value** |
| --- | --- | --- | --- |
| Age / 10 (years) | 2.52 | 1.91-3.33 | <0.001 |
| Prior cardiovascular disease^a^ | 2.36 | 1.10-5.08 | 0.03 |
| Phosphate / 0.1 (mmol/l) | 1.19 | 1.01-1.41 | 0.03 |

Studied by multivariate logistic regression.

Nagelkerke R^2^ = 0.32-0.33.

Abbreviations: OR, odds ratio; CI, confidence interval.

^a^Cardiovascular disease is defined as myocardial infarction, stroke, or vascular intervention.

**Supplementary Data 2** Cardiovascular events in patients with or without calcification

One out of 79 patients without abdominal aortic calcification (AAC) had a cardiovascular event (percutaneous treatment of peripheral arterial disease). The other 25 cardiovascular events occurred in the patients with AAC.

In univariate Cox regression presence of AAC (calcification score >0) was significantly associated with cardiovascular outcome: hazard ratio 10.2 (95% confidence interval 1.4-75.0), P-value 0.02.

We also performed propensity score matching. Forty nine matched pairs of patients with and without AAC were included in the analysis (data not shown). In four patients a cardiovascular event occurred, these were all patients with a calcification score >0.

**Reference:**

1. Cohen J (1968) Weighted kappa: nominal scale agreement with provision for scaled disagreement or partial credit. Psychological bulletin 70 (4):213-220
